# Social Exclusion in Health and Social Services Offered to Minority Patients: Do Racialization and Racism Play Roles?

**DOI:** 10.1093/geroni/igaa057.1849

**Published:** 2020-12-16

**Authors:** Sandra Torres

**Affiliations:** Uppsala University, Uppsala, Sweden

## Abstract

Cultural and ethnic differences stemming from migration are a source of social exclusion in old age. This topic is of concern in part because an increased migration flow coupled with growing anti-immigrants sentiments in much of the Western world can ignite social exclusion mechanisms even when unintended. Given these trends, we ask whether racism figures in research on ethno-cultural and racial older minorities. Thus, based on a scoping review of peer-reviewed articles published between 1998-2017 (n=336), this presentation asks if, and how, racialization and racism inform this research. In answering these questions, this presentation will argue that the role that racism plays as a social exclusion mechanism that affects older ethnic and racial minorities needs to be studied in a systematic fashion. Part of a symposium sponsored by the International Aging and Migration Interest Group.

